# Enhanced Nutrient Removal in A_2_N Effluent by Reclaimed Biochar Adsorption

**DOI:** 10.3390/ijerph19074016

**Published:** 2022-03-28

**Authors:** Peng Chen, Junkang Wu, Yue He, Yaping Zhang, Ran Yu, Xiwu Lu

**Affiliations:** 1 Department of Environmental Science and Engineering, School of Energy and Environment, Wuxi Engineering Research Center of Taihu Lake Water Environment, Southeast University, Nanjing 210096, China; 230149616@seu.edu.cn (P.C.); amflora@seu.edu.cn (Y.Z.); yuran@seu.edu.cn (R.Y.); 2 Department of Water Supply and Drainage Science and Engineering, College of Civil Engineering, Nanjing Forestry University, Nanjing 210037, China; 3 Nanjing Institute of Environmental Sciences, Ministry of Ecology and Environment, Nanjing 210042, China; heyue@nies.org

**Keywords:** nitrogen and phosphorus, biochar, activated sludge, adsorption, temperature, pH

## Abstract

The excessive nitrogen and phosphorus discharged into the water environment will cause water eutrophication and thus disrupt the water ecosystem and even exert biological toxicities. In this study, the absorption removal of nitrogen and phosphorus from the anaerobic tank in an anaerobic–anoxic/nitrifying system using four different kinds of biowaste-reclaimed biochars were investigated and compared. The effects of temperature and pH on nutrient adsorption removal were further investigated. The four kinds of biochar were successfully prepared and well characterized using a scanning electron microscope, fourier transform infrared spectroscopy, X-ray diffraction and Brunner−Emmet−Teller methods. Generally, there was no significant change in chemical oxygen demand (COD) and NH_4_^+^-N removal efficiencies when treated by the different biochars, while the activated sludge biochar (ASB) displayed the highest total phosphorus (TP) removal efficiency. The initial TP concentrations (<40 mg/L) displayed no remarkable effects on the TP adsorption removal, while the increase of temperature generally enhanced TP and NH_4_^+^-N adsorptions on the ASB. Besides, the increase of pH significantly promoted NH_4_^+^-N removal but depressed TP removal. Moreover, the adsorption process of TP by the ASB complies with the secondary kinetic model, suggesting the chemical precipitation and physical electrostatic interaction mechanisms of TP adsorption removal. However, the adsorption of NH_4_^+^-N conformed to the inner-particle diffusion model, indicating that the NH_4_^+^-N adsorption was mainly involved with pore diffusions in the particles.

## 1. Introduction

Nitrogen and phosphorus are essential nutritional sources for biological growth. However, the excessive nutritional salt content of nitrogen and phosphorus in water bodies will cause water quality pollution phenomenon, that is, water eutrophication [1,2,3]. It is caused by the imbalance of the input and output of nutrient and thus disrupt the water ecosystem balance. The quick reproduction of algae and phytoplankton, forming blooms and red tide in the surface water [4], will destroy the balance of dissolved oxygen in the water environment and disrupt the flow of material and energy in the system [1,2]. Oxytoxin will cause biological poisoning in the water and make the whole water ecosystem gradually perish.

The discharge of urban domestic sewage is the main source of nitrogen and phosphorus in natural water bodies. It is also an important reason for the eutrophication of the lake water. In recent decades, with the rapid economic development and the continuous improvement of the urbanization rate, the production of urban sewage has increased by 5–8% in China [5]. Currently, the nutrients in the wastewater are mainly removed using biological treatment methods by microorganisms [6]. However, the traditional biological nutrient removal technology has many deficiencies, such as the insufficient carbon source, the competition between phosphorus accumulating organisms and denitrifiers for carbon source, and the contradiction of sludge retention time while conducting simultaneous nitrogen and phosphorus removal [7,8]. In addition, phosphorus is considered a non-renewable and strategic resource. Therefore, it is of great significance to develop new processes to realize efficient nutrient removal as well as phosphorus recovery.

In view of the deficiencies of traditional biological wastewater treatment, a new process of the anaerobic–anoxic/nitrifying/induced crystallization (A_2_N-IC) recovery has been previously proposed by our research group [9,10]. This process can not only significantly improve the efficiency of nitrogen removal but also realize the effective recovery of phosphorus resources and reduce the energy consumption of sewage treatment [11]. However, the effect of induced crystallization is greatly influenced by the phosphorus concentration of the anaerobic pool [10]. The phosphorus with concentrations as low as 0.35 mg/L in the anaerobic pool was not well recovered when using induced crystallization methods. Compared with the chemical crystallization, the biochar adsorption method is expected to display more advantages while under the condition of low phosphorus concentrations [12]. The adsorption saturated biochar can also be used for soil improvement and increase of fertilization effect, to realize the resource utilization of nitrogen and phosphorus [13,14].

As the by-product and the biowaste during biological wastewater treatment, the urban sewage sludge has already exceeded 30 million tons in production in 2018 [5]. At present, sludge treatment methods mainly include landfill, land use, building materials utilization, and incineration [15]. It requires large investment and consumption of building materials during transportation, storage and treatment of sludge biowaste. The low-temperature heating transformations of activated sludge into biochar as soil modification can remarkably decrease the treatment costs [16,17]. Meanwhile, the sludge biochar can improve the physical and chemical properties of soil and improve soil ecology [18,19]. Thus, in this study, the sludge biowaste was considered as the potential biochar to adsorb the nutrients in the A_2_N effluent. The adsorption-saturated biochar containing N and P for soil modification were expected to be beneficial for crop growth and can increase the number of exchangeable cations in the soil and provide the element supply source [20,21].

The objective of this study is to compare the absorption removal of N and P from the anaerobic pool in A_2_N system using different kinds of biochar. The effects of temperature and pH on nutrient adsorption removal were further investigated, as well as the adsorption characteristics and mechanisms. The study mainly includes: (1) Preparation and characterization of four kinds of biochar and comparison of adsorption removal effect of N and P; (2) Adsorption dynamics and isotherm analysis of the selected biochar; and (3) Influences of different initial concentration, reaction time, reaction temperature and pH on N and P adsorption removal and the potential influence mechanisms.

## 2. Materials and Methods

### 2.1. Preparation of the Biochar

A three-stage reaction vacuum atmosphere furnace was used to prepare the biochar, and the temperature layer was stabilized in the middle section through a three-stage heating method to produce finer biochar. The raw materials used in the experiment were wheat straw, applewood branches, China fir branches and activated sludge from Nanjing Chengdong wastewater treatment plant (Capacity: 200,000 m^3^/d; Coordinates: 32°00′00″ N, 118°51′36″ E). The raw materials were dried in oven at 60 °C, then crushed to 2 mm using an LFP-800 crusher, and finally screened for the following experiments. Five grams of the pretreated crushing raw material was used for biochar manufacture in the tube furnace. The middle section of the tube furnace was blocked with asbestos prior to pyrolyzation with aeration of nitrogen gas at a rate of 0.5 L/min. The heating rate and retention time were maintained at 10 °C/min and 1 h. After the temperature dropped to 25 °C, the samples were withdrawn from the carbonization furnace and then ground for characterization.

### 2.2. Biochar Characterization

The structural properties of biochar surfaces were characterized using emission scanning electron microscope (SEM), fourier transform infrared (FTIR) spectroscopy, X-ray diffraction (XRD) and Brunner-Emmet-Teller (BET) measurements. After drying the four biochar samples and spraying gold, the biochar surface structure was observed using a high-resolution field emission scanning electron microscope (SEM, SU8010, Hitachi, Tokyo, Japan). The energy spectrometer EDS (Oxford 7582, Oxford Instruments, Concord, MA, USA) was used for the metal analysis on the biochar surface. The surface functional group of biochar samples were analyzed using a FTIR spectroscopy (Nicolet IS50, Thermo Scientific, Waltham, MA, USA). The Wavenumber was in the range of 4000–400 cm^−1^. The scanning times were performed at 32 with a resolution of 4 cm^−1^. The specific surface area and pore diameter of biochar were determined using a BET analyzer (BSD-PS1/2/4, BSD Instrument, Beijing, China). Biochar crystal analysis was performed using the XRD (X-ray diffraction). The crystal structures of the biochar powder samples were analyzed using a Brooke XRD instrument (Bruker D8 advance, Bruker, Germany). The ray wavelength, voltage, and current were set as 0.15406 nm, 40 kV, and 40 mA, respectively. The XRD peak was identified using the origin software.

### 2.3. Wastewater Contaminates Adsorption and Degradation Test

Synthetic wastewater simulating the effluent of the anaerobic tank in the A_2_N system was used for the adsorption and degradation test. The sodium acetate, potassium dihydrogen phosphate and ammonium bicarbonate were used for synthetic wastewater to reach a final chemical oxygen demand (COD) concentration of 50 mg/L, total phosphorus (TP) concentration of 30 mg/L and NH_4_^+^-N concentration of 30 mg/L. The details of the trace metals were available in our previous studies [9,10]. One hundred milliliters of the synthetic sewage was put into a 250 mL flask and the initial pH was adjusted to 7 using HCl/NaOH solutions. After adding 0.3 g of the four biochar, the flasks were shaken at 150 rpm at room temperature. Samples were taken at 9, 12, 15, 20, 25, 30, 40, 70, 130 and 250 min and then filtered using a 0.45 *µ*m filter for COD, TP and NH_4_^+^-N measurement.

### 2.4. Temperature and pH Influence Test

One hundred milliliters of wastewater containing 50 mg/L COD and different concentrations of nutrients (concentrations setup seen in Table 1) were taken for adsorption test under three different temperature conditions of 288 K, 298 K and 308 K, respectively. The initial pH of the solution was kept around 7. For pH test, the initial COD, NH_4_^+^-N and TP concentrations were set as 50 mg/L, 30 mg/L, and 30 mg/L, respectively. The initial pH of each reaction system adjusted by HCl or NaOH solutions was kept as 2, 3, 4, 5, 6, 7, 8, 9, 10, 11 and 12, respectively. A 0.3 g of activated sludge biochar (ASB) was added into each 250 mL flask containing 100 mL wastewater for shaking at 150 rpm. After 4 h and 8 h of reaction, the NH_4_^+^-N and TP concentrations were measured.

### 2.5. Chemical Analysis

COD, NH_4_^+^-N, and TP concentrations were determined according to the standard methods [22]. pH was measured using a pH meter (HACH SC200, HACH, Loveland, CO, USA).

### 2.6. Analysis of Adsorption Kinetics and Adsorption Isotherm

The Pseudo-first order kinetic equations, Pseudo-second order kinetic and internal diffusion models [23,24,25] were applied for curve fitting of adsorption kinetics of TP and NH_4_^+^-N by the ASB. The fitting equations were as follows:

The first-order kinetic equation:

(1) $$\frac{dQ_{t}}{dt}=K_{1}\left(Q_{e}-Q_{t}\right)$$

The second-order kinetic equation:

(2) $$\frac{dQ_{t}}{dt}=K_{2}{\left(Q_{e}-Q_{t}\right)}^{2}$$

Internal diffusion model:

(3) $$Q_{t}=K_{3}t^{0.5}+b$$

where: *Q_e_*—the equilibrium adsorption capacity, mg/g;

*Q_t_*—the adsorption capacity at time t, mg/g;

*K*_1_—the first adsorption rate constant;

*K*_2_—the second adsorption rate constant;

*K*_3_—the inter-diffusion constant between particles.

The calculation formula of the adsorption quantity *Q* is:

(4) $$Q=\frac{\left(C_{0}-Ce\right)V}{m}$$

where: *C*_0_—the initial TP or NH_4_^+^-N concentration, mg/L;

*C_e_*—the TP or NH_4_^+^-N concentration when reaching adsorption equilibrium, mg/L;

*V*—the reaction volume, L;

*m*—the added biochar mass, g.

The Langmuir and Freundlich equations were applied for curve fitting after 4 h and 8 h adsorption reaction shown as follows:

The Langmuir isothermal equation:

(5) $$Q=\frac{K_{L\;}Q_{m}C_{e}}{1+K_{L\;}C_{e}}$$

The Freundlich isothermal equation:

(6) $$Q=k_{F}c_{e}^{n}$$

where: *Q*—the adsorption capacity, mg/g;

*C_e_*—the TP or NH_4_^+^-N concentration when reaching equilibrium, mg/L;

*Q_m_*—the maximum equilibrium adsorption capacity, mg/g;

*K_L_*—the Langmuir constant;

*K_F_*—Freundlich constant.

## 3. Results and Discussions

### 3.1. Biochar Characteristics

#### 3.1.1. SEM-EDS

**Figure 1:**
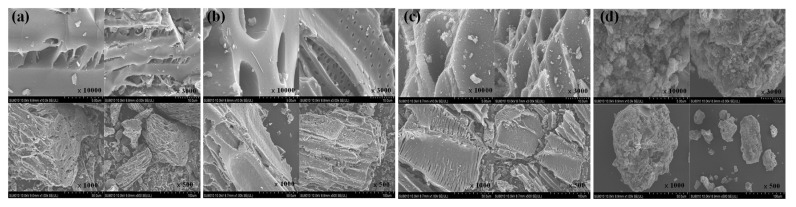
SEM morphology of four kinds of biochar: (**a**) Straw biochar; (**b**) Applewood biochar; (**c**) China fir biochar; (**d**) Activated sludge biochar.

SEM imaging with four different scanning ranges of the prepared biochars was shown in Figure 1. It can be seen that the surface morphology of the ASB is significantly different from that of the three other plant biochars. The surface morphology and structure of the biochars are highly related to the characteristics of the raw materials. The straw biochar (STB), applewood biochar (AWB), and China fir biochar (CFB) displayed a fiber structure on the surface, which accorded with the structure of the raw materials [26]. Uneven distribution of pore size and irregular cracks were observed on the STB surface (Figure 1a). The AWB displayed a smooth surface and parallel round hole shape structure (Figure 1b). In addition, the CFB had a dense pore distribution and a large pore size, and an obvious layered structure was determined (Figure 1c). Instead of having the fiber structure, the ASB presented a relatively large and compact particulate structure, probably due to the carbonization of organic matter in the sludge (Figure 1d). Moreover, the ASB surface is rough with the presence of irregular granular carbon, and the pore distribution was disordered. This might be due to the carbonization and excessive fragmentation of the organic matter in the sludge (Figure 1d). Cheng et al. have also reported similar ASB characteristics as observed in this study [27].

**Figure 2:**
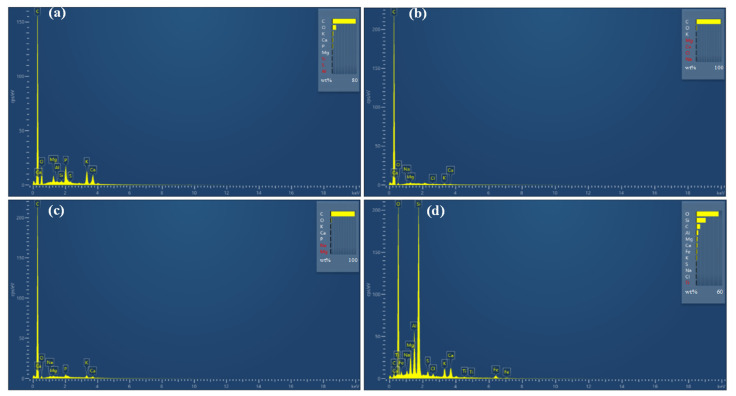
EDS spectrum of four kinds of biochar: (**a**) Straw biochar; (**b**) Applewood biochar; (**c**) China fir biochar; (**d**) Activated sludge biochar.

The results of the energy profiles of the four different biochars are shown in Figure 2 to obtain the element components in the biochar. The STB surface was rich in carbon and oxygen elements, with atomic mass scores of 77.3% and 12.9%, respectively, in addition to the elements of P, K, Ca, and Mg (Figure 2a). Instead, the elements on the AWB surface were not as rich as those on the STB. The carbon element amount dominated in AWB as high as 96.1%, followed by the oxygen element accounting for 2.9% (Figure 2b). The element components in the CFB were similar to the AWB (Figure 2b,c). In addition, the ASB had the lowest carbon content as low as 10% when compared with the other three biochars but was the one most rich in element types—as many as 12 species (Figure 2d). The most abundant element in the ASB was the oxygen element, accounting for 50%, followed by silicon element (20%). Moreover, there were several metal elements with low abundance including Mg, Al, K, Ca, Fe, etc. Many studies have reported that the carbon and potassium content in non-cellulosic biochar are lower than that in cellulose biochar, while Ca, Mg, Al and other elements are higher than that in cellulose biochar [28,29,30], which was in accordance with the EDS results obtained in our study.

#### 3.1.2. FTIR

**Figure 3:**
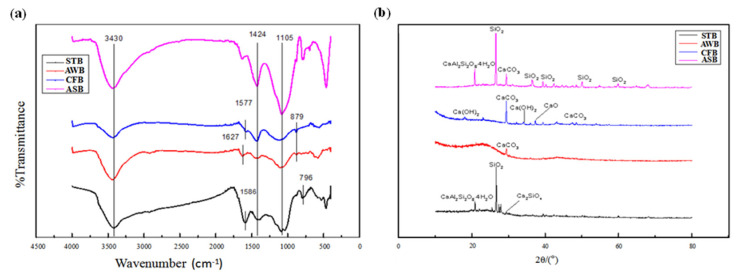
Fourier transform infrared (**a**) and X-ray diffraction (**b**) spectra of the four biochars.

The functional group profiling of the biocarbon surface was obtained by FTIR analysis as shown in Figure 3. It can be seen that the four biochars have similar characteristic absorption peaks and thus similar functional groups on the surface. The AWB and CFB show a highly consistent absorption peak, indicating that the raw material composition of the AWB and CFB was very similar. There are three obvious characteristic peaks in the spectrum with wavenumbers of 3430, 1424 and 1105 cm^−1^ (Figure 3a). The ASB had the largest adsorption peak at 3430 cm^−1^, which is caused by O-H telescopic vibration indicating the phenol and alcohol hydroxyl functional group [31]. The absorption peak at 1424 cm^−1^ is caused by C=C stretching vibration, and the peak near 1105 cm^−1^ is mainly attributed to C–O–C antisymmetric stretching vibration [32,33]. In addition, the peak at 1586 cm^−1^ is due to the aromatized C=C stretching vibration [34] and multiple aromatic C–H deformation vibration was also observed at the peaks of 600–800 cm^−1^ [35], including 796 and 879 cm^−1^ peaks. Therefore, the presence of aromatic compounds was available in all four biochars. The STB and CFB have also been characterized to contain hydroxyl group, aromatic structure and carboxyl group but without ether functional groups by FTIR spectroscopy analysis [36], which is consistent with the main functional groups obtained in this study. In summary, the four biochars had the hydroxy and ether functional groups and aromatic structures, and the ASB was minimally aromatized.

#### 3.1.3. XRD

The X-ray diffraction profiles of four different biochars were shown in Figure 3b. It can be seen that the STB and the ASB had an obvious peak at 26.6° and the AWB and CFB had a peak at 29.4° (Figure 3b). The highest peak at 26.6° in the ASB has been also documented as SiO_2_, as well as the presence of metal oxides, including CaCO_3_, CaO and MgO [37,38], which was similar to the XRD and EDS results in this study. In addition, the strength of the STB and the ASB diffraction peak is relatively high than the others, indicating the high crystallization performance of the STB and the ASB. The high crystallization of biochar has been reported to display better adsorption performance and high stability [39]. Therefore, the ASB was expected to exert the best adsorption performance. In addition, there are multiple diffraction peaks occurring in the CFB and ASB between 40° and 70°, indicating that the ASB and CFB surfaces are mineralized with a certain amount of mineral composition.

#### 3.1.4. BET

**Figure 4:**
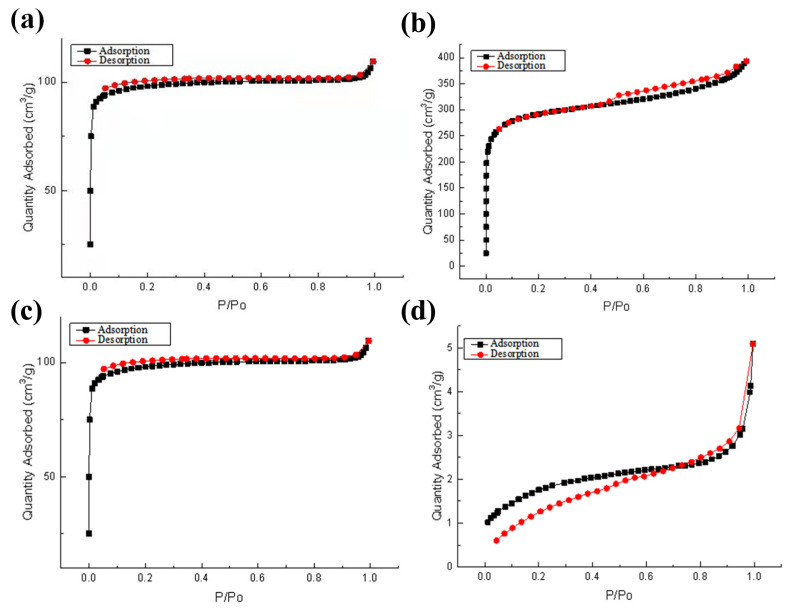
N_2_ adsorption–desorption curve of four kinds of biochar: (**a**) Straw biochar; (**b**) Applewood biochar; (**c**) China fir biochar; (**d**) Activated sludge biochar.

The specific surface area and porosity of biochar are critical for its adsorption properties, which was determined using BET method in this study. The N_2_ adsorption–deattachment curves of the four biochars were shown in Figure 4. According to the International Union of Pure and Applied Chemistry (IUPAC) classification, the adsorption–deattachment curves of the ASB and STB belong to the II type curve, while those of the CFB and AWB belong to the type I curve. In the CFB and AWB, there are obvious microporous monolayer adsorption features in the low relative pressure region, and an extension at the relative pressures of 0.1–0.3 and 0.5–0.9 was observed. It can be inferred from the curve that the pore of the AWB and the CFB was mainly slit shaped. In addition, the adsorption capacity rapidly increased under low relative pressure and thus caused a convex shape of the curve in the ASB and STB, probably due to the strong interaction between the adsorbate and the biochar surface.

The specific surface area and pore size of four kinds of biochar were shown in Table 2. The average pore diameter of the four biochars was similar, in the range of 4.56–6.49 nm (Table 2). The order of the specific surface area of the four biochars is: CFB >> AWB > ASB >> STB, and the specific surface area size is consistent with the SEM imaging results. The specific surface area of biochar is related to the type of raw materials and the volatile components contained. During the preparation of biochar, the volatilization facilitates the formation of pore structure, and cellulosic biomass has rich internal pore [40,41], which might result in the high specific surface area of CFB and AWB. The specific surface area of CFB reached 1116.13 m^2^/g and had a pore capacity of 0.338 cm^3^/g, indicating that the CFB might probably display good physical adsorption capacity.

### 3.2. Adsorption Removal of COD, TP and NH_4_^+^-N

**Figure 5:**
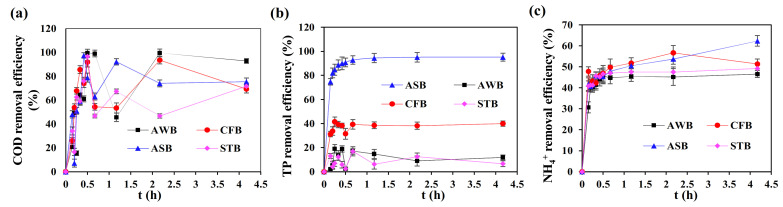
Adsorption effect of four kinds of biochar on: (**a**) COD; (**b**) TP and (**c**) NH_4_^+^-N.

Generally, there was no significant change in COD and NH_4_^+^-N variations between the four biochar tests. As shown in Figure 5a,c, the COD and NH_4_^+^-N removal efficiencies all rapidly increased within 0.5 h. With the prolonged reaction time (0.5–4 h), there was an obvious fluctuation in COD removal in all the adsorption tests, while the NH_4_^+^-N removal rates gradually reached stable. The fluctuation in COD removal might be attributed to the biochar dissolution and COD release from the biochar. Odedishemi et al. [42] determined that the dissolved organic carbon release from the biochar in a constructed wetland and the COD could actually be utilized by the microorganisms. The highest COD removal efficiencies by the AWB, CFB, ASB and STB were achieved at 99.6%, 91.8%, 97.5% and 96.6%, respectively (Figure 5a). After 4 h of reaction, the ASB displayed a slightly higher NH_4_^+^-N removal efficiency of 62.4% than the other biochars with the NH_4_^+^-N removal between 46.6–51.3% (Figure 5c).

The TP adsorption effect varied significantly from different biochars. After 0.25 h of reaction, the TP removal efficiencies achieved by the AWB, CFB, STB and ASB rapidly reached at 19.1%, 41.6%, 12.5% and 84.7%, respectively (Figure 5b). After that, the TP removal gradually kept stable. In addition, the ASB exerted the highest TP adsorption removal rate of 95.1%, significantly higher than the AWB (11.9%), CFB (40.0%) and STB (6.6%) (Figure 5b). sludge biochar shows a great advantage in TP adsorption. Thus, the ASB displayed higher TP removal efficiency than the other three biochars and was expected to play a critical role in enhanced P removal in A_2_N-IC system.

The mechanism of biochar adsorption of phosphate is well reported to include multiple reactions [24,43]. The biochar surface with a positive charge could interact with the phosphate anion by electrostatic interaction, which generally occurs at the beginning of the adsorption reaction [24]. In addition, the metal and the oxides on the biochar surface could react with the phosphate in the aqueous solution through precipitation to form stabilized precipitates [43]. Moreover, multivalent cations (e.g., Ca, Mg, Fe, and Al) in the biochar can chelate with phosphate to form composite minerals using oxygen-containing functional groups as bridge bonds. The abundant metal elements in the ASB by EDS analysis and the obtained phenol and alcohol oxhydryl functional groups by FTIR analysis previously further supported the potential precipitation and complexation reactions mechanisms of TP removal by the ASB. Therefore, the chemical adsorption reaction including electrostatic adsorption, surface precipitation and complex cooperation contributed to the excellent TP removal performances of the ASB in this study. The ASB will be selected for the following temperature and pH influencing test.

### 3.3. Effects of Temperature on Adsorption-Removal Efficiency

**Figure 6:**
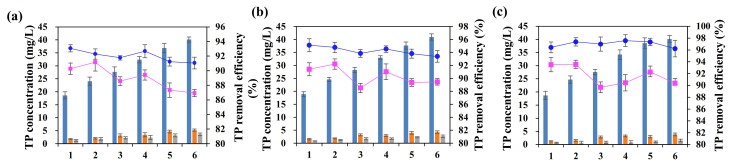
TP removal at different temperatures: (**a**) 288 K; (**b**) 298 K; (**c**) 308 K.

Generally, the TP concentrations displayed a similar variation trend when treated by the ASB at different temperatures as seen in Figure 6. After 4 h and 8 h of reaction, the TP was all remarkably removed by more than 86% and 90%, respectively, at all the three temperatures (Figure 6a–c), which indicated that the adsorption equilibrium might have been probably reached within 4 h. The increase of initial TP concentrations from 18 to 40 mg/L slightly depressed the TP removal efficiencies by 3.7%, 2.1% and 0.3% under 288 K, 298 K and 308 K conditions, respectively after 8 h of reaction (Figure 6). It is inferred that the initial phosphorus concentration below 40 mg/L had few effects on the adsorption efficiency. The increase of temperature generally enhanced the TP adsorptions on the ASB. The TP removal rate significantly increased from 92.2 ± 0.7% to 97.0 ± 0.6% when the temperature rised from 288 K to 308 K (Figure 6a–c).

**Figure 7:**
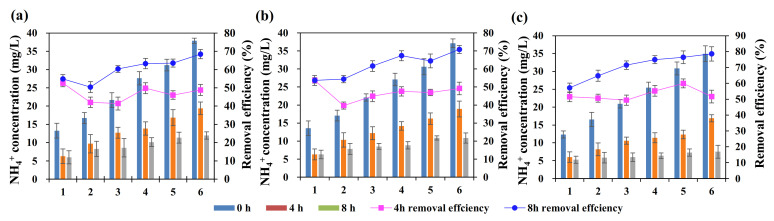
NH_4_^+^-N removal efficiency at different temperatures: (**a**) 288 K; (**b**) 298 K; (**c**) 308 K.

The NH_4_^+^-N removal remarkably varied from the initial concentrations, the reaction time and the temperatures, as seen in Figure 7. The NH_4_^+^-N removal was kept stable at 48.2 ± 3.9% after 4 h of reaction regardless of the initial NH_4_^+^-N concentrations and the temperatures. Nevertheless, the NH_4_^+^-N removal rate increased by 36.9 ± 5.0% after 8 h of reaction and displayed a significantly positive relation to the initial NH_4_^+^-N concentrations at all three temperatures. Therefore, the NH_4_^+^-N reached the adsorption equilibrium only after 8 h of reaction, longer than that than when the TP reached adsorption equilibrium. In addition, the increase of temperature from 288 K to 308 K dramatically enhanced the NH_4_^+^-N removal from 61.2 ± 6.7% to 73.2 ± 5.4%, suggesting that the high temperature is conducive to the adsorption reaction and the removal of ammonia nitrogen.

### 3.4. Effects of pH on Adsorption-removal Efficiency

**Figure 8:**
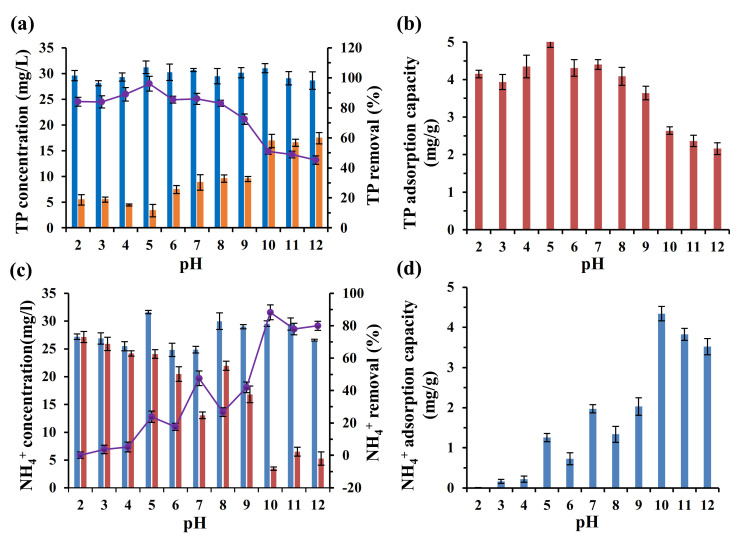
Effect of pH on the removal rate and the adsorption capacity of TP (**a**,**b**) and NH_4_^+^-N (**c**,**d**), respectively.

The effect of initial pH on the TP adsorption removal is shown in Figure 8a,b. The adsorption removal of TP in wastewater firstly increased from 84.1% to 96.2% when pH increased from 2 to 5, then gradually decreased, and a significant reduction in the removal efficiency to around 45.2% was observed when pH higher than 9. The corresponding adsorption capacity increased from 4.2 mg/g to 5.1 mg/g, and then decreased only to 2.2 mg/g. Therefore, the increase of pH under faintly acid conditions would promote TP adsorption by ASB, while the high alkalinity might exert notable adverse effects on TP adsorption.

The form of P in the water and biochar surface charge under different pH conditions would be probably responsible for the varying adsorption efficiencies. Generally, P present in the water is a form of PO_4_^3−^, HPO_4_^2^ and H_2_PO_4_^−^, with the equilibrium constants of 2.15, 7.20 and 12.33, respectively [44]. P is mainly in the form of anionic H_2_PO_4_^−^ with pH at 2–7. Meanwhile, the pH value in the wastewater will affect the ASB surface charge [45]. When the pH value in wastewater is less than zero-point charge (zeta potential), the protonation of ASB chemicals could lead to a positive charge on the surface. The zero-point charge (zeta potential) of the ASB was measured as 5.46. The adsorption removal was, therefore, expected to increase due to the electrostatic attraction between H_2_PO_4_^−^ and OH group (Figure 3a) on the ASB surface confirmed by FTIR analysis above, and the adsorption performance gradually decreased when pH was higher than 5. In addition, when pH increased higher to 10, P is mainly in the form of HPO_4_^2-^ and OH^-^ would be abundant in the wastewater^-^, which might probably compete with HPO_4_^2-^ for adsorption on the positively charged ASB surface.

The effect of initial pH on the adsorption removal of NH_4_^+^-N is shown in Figure 8c,d. When the pH increased from 2 to 12, the adsorption removal of NH_4_^+^-N by ASB significantly increased by around 80%. It has been well-known that the ammonia nitrogen in the wastewater is mainly in the form of NH_4_^+^ when the solution is under acidic conditions [46]. Thus, the positively charged NH_4_^+^ would probably repel to ASB surface with a positive charge and compromised the adsorption effect. With the decrease of H^+^ concentration and increase of OH^-^ concentration as pH rising, ammonia nitrogen in the wastewater began to be in the form of free NH_3_ and the electrostatic attraction between NH_3_ and ASB gradually enhanced, which finally resulted in the high adsorption capacity of NH_4_^+^-N. The adsorption capacity of ammonia nitrogen increased from 1.97 mg/g to 4.34 mg/g by 120% when pH ranging from 7 to 10.

### 3.5. Adsorption Dynamics and Adsorption Isotherm

**Figure 9:**
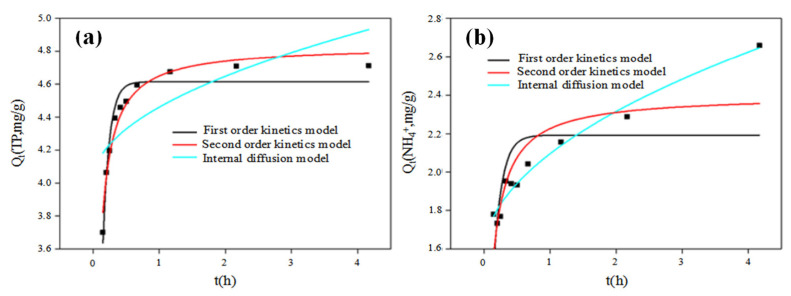
Adsorption kinetics fitting of TP (**a**) and NH_4_^+^-N (**b**) by the activated sludge biochar.

The TP adsorption process was relatively more in line with the Pseudo-second order and Pseudo-first order model, with the correlation coefficient of 0.9623 and 0.9351, respectively, which is significantly higher than that (0.4716) of the internal diffusion model (Table 3 and Figure 9). The Pseudo-first order kinetic model was suitable for fitting at the beginning of the adsorption reaction rather than the substrate transmission period. The Pseudo-second kinetic model includes liquid membrane diffusion, internal diffusion and surface adsorption, and the adsorption process is mainly controlled by chemical action, such as electron sharing or electron transfer and broken chemical bond and generation [47,48]. As discussed above, the mechanisms of TP adsorption by the ASB mainly included chemical precipitation with metal oxide and electrostatic interaction on the surface [43,49]. The observed abundant metal elements (e.g., Ca, Mg and Fe) and oxygen-contained groups (e.g., phenol and alcohol hydroxyl group) on the ASB surface further supported the proposed adsorption removal mechanisms. In summary, the Pseudo-second kinetic model was expected to better describe the TP adsorption removal process by the ASB.

The adsorption process of NH_4_^+^ onto the ASB is mainly consistent with the intra-particle diffusion model with a correlation coefficient of 0.9653 (Table 3 and Figure 9b), significantly higher than that of the Pseudo-first or Pseudo-second kinetic model, indicating that the adsorption mechanisms were involved with physical diffusion from the particle surface through the internal pore. The porous structure of the ASB with compact and irregular granular particles determined by SEM imaging in Figure 1d might support the intraparticle diffusion process during the NH_4_^+^ adsorption.

**Figure 10:**
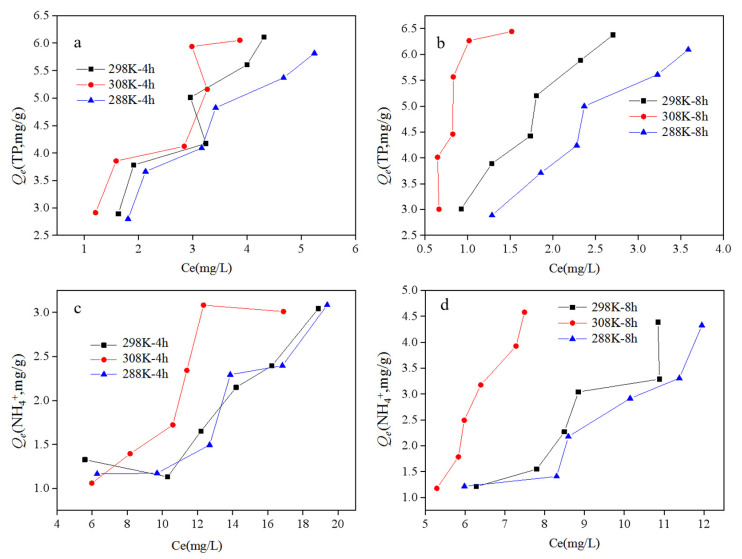
Adsorption isotherm curve of TP (**a**,**b**) and NH_4_^+^-N (**c**,**d**) by activated sludge biochar after 4 h and 8 h of reaction, respectively.

The Langmuir and Freundlich models [50] were finally applied for fitting the adsorption isotherm curve of TP and NH_4_^+^-N, as shown in Figure 10 and Table 4. Generally, an obvious S-type curve of TP and NH_4_^+^-N adsorption was observed, suggesting that at least two binding energy levels [51] occurred during the adsorption by the ASB. The Langmuir isothermal equation usually shows that the adsorbent is single-layer distributed on the adsorbent surface, while the Freundlich isothermal equation describes the multi-layer uneven adsorption and the adsorption capacity will increase with the increasing concentration [50]. In this study, similar correlation coefficients were obtained when fitting using Langmuir and Freundlich models (Table 4), which revealed that TP adsorption by dirty peat was probably a result of multiple processes, including both monolayer adsorption and multi-layer adsorption processes. In contrast, the Freundlich model could be used for fitting the curve of NH_4_^+^-N adsorption rather than the Langmuir model except for the 3 h of adsorption test at 35 °C (Table 4). It indicated that the adsorption of NH_4_^+^-N is mainly a process of multi-layer adsorption.

## 4. Conclusions

In this study, the STB, AWB, CFB and ASB were successfully prepared and well-characterized. Generally, no significant change in COD and NH_4_^+^-N variations were determined when treated by different biochars, while the ASB showed higher TP removal efficiencies than the others. The variations of initial phosphorus concentration did not remarkably affect the adsorption efficiency. In addition, the increase of temperature generally promoted the TP and NH_4_^+^-N adsorptions on the ASB. the increase of pH notably enhanced NH_4_^+^-N removal but decreased TP adsorption removal. What is more, the adsorption process of TP by the ASB was in line with the secondary kinetic model, reflecting the chemical precipitation and physical electrostatic interaction mechanisms of TP adsorption removal. In contrast, the process of NH_4_^+^-N accorded with the inner-particle diffusion model, suggesting that the NH_4_^+^-N adsorption process was mainly associated with pore diffusions in the particles.

## Figures and Tables

**Table 1:** Concentrations Setup of NH_4_^+^-N and TP Adsorption Test.

Name	Test 1	Test 2	Test 3	Test 4	Test 5	Test 6
NH_4_^+^ adsorption test						
NH_4_^+^-N (mg/L)	30	30	30	30	30	30
TP (mg/L)	14	18	22	26	30	34
TP adsorption test						
NH_4_^+^-N (mg/L)	15	20	25	30	35	40
TP (mg/L)	30	30	30	30	30	30

Notes: TP: Total phosphorus.

**Table 2:** Specific Surface Area and Pore Size of Four Kinds of Biochar.

Biochar	Specific Surface Area (m^2^/g)	Micropore Surface Area (m^2^/g)	Total Pore Volume (cm^3^/g)	Micropore Volume (cm^3^/g)	Average Pore Size (nm)	Iodine Sorption Value (mg/g)
STB	6.3958	0.7819	0.0048	0.0002	6.2728	287
AWB	391.0390	353.8750	0.1583	0.1353	4.7700	285
CFB	1116.1316	860.9206	0.5726	0.3384	4.5637	1115
ASB	123.3406	60.6806	0.0995	0.0256	6.4946	187

Notes: STB: Straw biochar; AWB: Applewood biochar; CFB: China fir biochar; ASB: Activated sludge biochar.

**Table 3:** Specific surface area and pore size of four kinds of biochar.

	Pseudo-First Order			Pseudo-Second Order			Intraparticle Diffusion		
	*K*_1_ (h^−1^)	*Q_e_*_1_ (mg/g)	*R^2^*	*K*_2_ (g/(mg·h))	*Q_e_*_2_ (mg/g)	*R^2^*	*K*_3_ (mg/(g·h))	*B* (mg/g)	*R* ^2^
TP	10.3352	4.6149	0.9351	0.0866	4.8348	0.9623	0.4532	4.0074	0.4716
NH_4_^+^-N	2.1923	7.9165	0.3579	5.3135	2.4004	0.7125	0.5297	1.5658	0.9653

**Table 4:** Fitting parameters by Langmuir and Freundlich adsorption isotherms.

	Temperature	Time	Langmuir				Freundlich		
			*Q*_m_ (mg g^−1^)	*K*_L_ (L/mg)	*R*_L_	*R* ^2^	*K*_F_ (mg/g)	*N*	*R* ^2^
TP	288 K	4 h	11.2226	0.2020	0.2102	0.9468	2.1508	0.6025	0.9414
		8 h	16.4065	0.1643	0.2465	0.9589	2.4477	0.7186	0.9554
	298 K	4 h	13.3081	0.1821	0.2244	0.8408	16.0666	0.2286	0.8481
		8 h	15.9048	0.2486	0.1749	0.9658	3.2284	0.6965	0.9648
	308 K	4 h	10.3890	0.3319	0.1389	0.7406	2.7344	0.5730	0.7482
		8 h	14.8483	0.5657	0.0864	0.6320	5.2987	0.6401	0.5962
NH_4_^+^-N	288 K	4 h	-	-	-	-	0.1075	1.1174	0.8501
		8 h	-	-	-	-	0.0193	2.1540	0.9089
	298 K	4 h	-	-	-	-	0.1409	1.0227	0.7638
		8 h	-	-	-	-	0.0250	2.1166	0.8118
	308 K	4 h	68.5823	0.0029	0.9651	0.7493	0.1913	1.0048	0.7482
		8 h	-	-	-	-	0.0074	3.1919	0.9271

## Data Availability

Data is contained within the article.
